# Die Menstruation auf YouTube, Instagram und TikTok: Eine Inhalts- und Qualitätsanalyse

**DOI:** 10.1007/s00103-026-04213-x

**Published:** 2026-03-18

**Authors:** Nicola Döring, Anastasiia Shevtsova, Claudia Schumann-Doermer

**Affiliations:** 1 Institut für Medien und Kommunikationswissenschaft (IfMK), Technische Universität Ilmenau, Ehrenbergstraße 29, 98693 Ilmenau, Deutschland; 2 Deutsche Gesellschaft für psychosomatische Frauenheilkunde und Geburtshilfe (DGPFG), Dresden, Deutschland

**Keywords:** Monatsblutung, Gesundheitsinformationen, mDISCERN-Index, Periode, Soziale Medien, Menstrual flow, Health information, mDISCERN index, Period, Social media

## Abstract

**Hintergrund:**

Jugendliche und Erwachsene suchen Informationen zur Menstruation zunehmend in sozialen Medien. Vor diesem Hintergrund war es Ziel der vorliegenden Studie, erstmals Inhalte und Qualität deutschsprachiger Menstruationsvideos auf YouTube, Instagram und TikTok zu untersuchen. Beantwortet werden sollen Forschungsfragen (F) zu Anbietertypen (F1), Inhalten (F2) und Qualität der Menstruationsvideos (F3) sowie zu Publikumsreaktionen (F4).

**Methoden:**

Im Jahr 2024 wurde eine Stichprobe von *N* = 500 populären Menstruationsvideos von YouTube (150), Instagram (150) und TikTok (200) gezogen. Pro Video gingen die maximal 20 meistgelikten themenbezogenen Kommentare ein (*N* = 6314). Die Videos und Kommentare wurden mittels reliabilitätsgeprüfter Codebücher analysiert. Die Datenanalyse erfolgte mit R. Die Studie ist präregistriert und alle Daten, Materialien und Analyseskripte sind öffentlich verfügbar.

**Ergebnisse:**

Die untersuchten Menstruationsvideos stammten überwiegend von Gesundheitslaien (42 %) und seltener von Gesundheitsprofis (17 %; F1). Die Darstellung der Menstruation war überwiegend neutral (52 %); einseitig negativ (15 %) oder positiv (13 %) gefärbte Darstellungen waren seltener. Inhaltlich drehten sich die Videos vor allem um das Menstruationserleben (z. B. Schmerzen) sowie um den praktischen Umgang mit der Blutung (z. B. Menstruationsprodukte; F2). Gemäß Qualitätskriterien für evidenzbasierte Gesundheitsinformationen zeigten sich deutliche Defizite (F3). Die Kommentarspalten wurden vom Publikum genutzt, um persönliche Erfahrungen zu teilen und Fragen zu stellen (F4).

**Diskussion:**

Weitere Forschung sowie Praxismaßnahmen sind notwendig, um die Qualität von Social-Media-Videos zur Menstruation besser einschätzen und optimieren zu können.

## Abstract (en)

**Background:**

Young people and adults are increasingly searching for information about menstruation on social media. Against this background, the present study aimed, for the first time, to examine the content and quality of German-language menstruation videos on YouTube, Instagram, and TikTok. The study addresses research questions (RQ) on content provider types (RQ1), content (RQ2), and the quality of menstruation videos (RQ3) as well as audience reactions (RQ4).

**Methods:**

In 2024, a sample of *N* = 500 popular menstruation videos was drawn from YouTube (150), Instagram (150), and TikTok (200). For each video, up to 20 of the most liked topic-related audience comments were included (*N* = 6314). Videos and comments were analyzed using reliability-tested codebooks. Data analysis was performed with R. The study is preregistered, and all data, materials, and analysis scripts are publicly available.

**Results:**

The included menstruation videos predominantly originated from laypersons (42%) and less frequently from healthcare professionals (17%; RQ1). The portrayal of menstruation was mostly neutral (52%); negative (15%) or positive (13%) depictions were less frequent. Content-wise, the videos primarily addressed the experience of menstruation (e.g., pain) and practical management of bleeding (e.g., menstrual products; RQ2). According to quality criteria for evidence-based health information, substantial deficits were observed (RQ3). Comment sections were used by viewers to share personal experiences and to ask questions (RQ4).

**Discussion:**

Future research and practical measures are necessary to further assess and improve the quality of social media videos about menstruation.

## Hintergrund

Die *Menstruation* (auch: Monatsblutung; Regelblutung) bezeichnet die bei nicht eingetretener Schwangerschaft typischerweise monatlich stattfindende Abstoßung der funktionellen Schicht der Gebärmutterschleimhaut, die mit einer mehrtägigen Blutung aus der Gebärmutter nach außen über die Vagina einhergeht [1]. Zwischen erster Menstruation (Menarche) im Alter von durchschnittlich etwa 13 Jahren und letzter Menstruation (Menopause) im Alter von etwa 53 Jahren finden im Laufe des Lebens einer Frau bzw. einer Person mit Gebärmutter^1^ rund 450 Blutungen statt [2, 3].

Die Menstruation ist ein wichtiges Gesundheitsthema, da sie bei einer nennenswerten Zahl von Betroffenen mit unterschiedlichen Beschwerden [4, 5] und auch mit klinischen Störungen einhergeht, die entsprechende medizinische Versorgung benötigen [6, 7]. Hier wird zuweilen beklagt, dass entsprechende Beschwerden (z. B. im Rahmen des prämenstruellen Syndroms (PMS) oder von Schmerzen während der Menstruation) nicht ernst genommen und dass klinisch relevante prämenstruelle und menstruelle Störungen sowie zyklusabhängige Erkrankungen (z. B. Endometriose) zuweilen zu spät erkannt und unzureichend behandelt werden [3].

Doch die Menstruation ist nicht nur ein biologisches Geschehen, das mit der menschlichen Fortpflanzungsfähigkeit und spezifischen gesundheitlichen Problemen zusammenhängt. Sie hat weitreichende psychische und soziale Dimensionen, die letztlich auch Fragen der Frauenrechte und Geschlechtergleichberechtigung betreffen [8]. So unterliegt die Monatsblutung – je nach kulturellem Kontext – mehr oder minder starker Tabuisierung und Stigmatisierung in dem Sinne, dass sie als „unrein“, „schmutzig“, „eklig“ und „peinlich“ gilt, sodass Mädchen und Frauen gezwungen sind, die Menstruation vor ihrem Umfeld zu verbergen und sich während der Blutung aus sozialen Aktivitäten und Gemeinschaften zurückzuziehen [3]. Die Tatsache, dass ein mit dem weiblichen Körper verbundener natürlicher Prozess, der die meisten Frauen über mehrere Jahrzehnte ihres Lebens regelmäßig begleitet, so negativ konnotiert ist, kann sich ungünstig auf ihr Körper- und Selbstbild auswirken. Auch das öffentliche Bild von Frauen wird von der Menstruation mitgeprägt, etwa durch das Klischee der „emotional labilen“, „hysterischen“ und „leistungsschwachen“ menstruierenden Frau [8].

Zugänglichkeit, Sicherheit und Kosten von Menstruationsprodukten sind ein internationales Problem. Die Lebenszeitkosten für Menstruationsprodukte werden auf rund 7000–17.000 € geschätzt, was Menstruationsarmut (engl. „period poverty“) begünstigen kann [3, 9]. Es bedurfte zahlreicher Kampagnen aus dem Menstruationsaktivismus, um politisch zu erreichen, dass in Deutschland seit dem 01.01.2020 für Menstruationsprodukte ein ermäßigter Mehrwertsteuersatz erhoben wird, da sie nun nicht mehr als Luxusartikel gelten, sondern zum Grundbedarf gezählt werden.

Mit *Menstruationsgesundheit* (engl. „menstrual health“) ist mehr gemeint als die Abwesenheit von menstruellen Störungen und Beschwerden. Vielmehr geht es um ein ganzheitliches physisches, psychisches und soziales Wohlbefinden rund um die Menstruation [1]. Die Weltgesundheitsorganisation (WHO) hat im Jahr 2022 die Menstruationsgesundheit offiziell als vordringliches globales Gesundheitsthema anerkannt und auch Menstruationsrechte formuliert [10].

Eine wichtige Voraussetzung für die Sicherung der Menstruationsgesundheit ist umfassende Aufklärung. Denn nur eine offene und sachgerechte Kommunikation über die Menstruation kann tradierter Tabuisierung und Stigmatisierung entgegenwirken, notwendige Informationen zu den vielfältigen Dimensionen des Themas liefern und Menstruierende in ihrem Wohlbefinden unterstützen.

Jugendliche in Deutschland berichten mehrheitlich, dass im Sexualkundeunterricht in der Schule im Zusammenhang mit Fortpflanzung und Eisprung auch „die Regel“ als Thema vorgekommen ist [11], gleichzeitig sagen jedes 4. Mädchen und jeder 5. Junge, dass sie mehr darüber wissen wollen [12]. Denn jenseits der biologischen Betrachtung im Kontext von Fruchtbarkeit werden psychosoziale Aspekte der Menstruation, etwa das Erleben von Schmerz und Scham, sexistische Periodenwitze, die Anwendung von unterschiedlichen Menstruationsprodukten, sportliche Leistungsfähigkeit während der Tage oder Vereinbarkeit von Menstruation mit sexueller Aktivität im Biologie- und Sexualkundeunterricht meist nicht behandelt – obwohl das durchaus Fragen sind, die Mädchen, Jungen und nichtbinäre Jugendliche interessieren [1]. Junge Menschen greifen daher für Sexualaufklärung, einschließlich Menstruationsaufklärung, gern auf das Internet zurück, wo sie Webseiten zum Thema finden oder auf den populären Social-Media-Plattformen Posts und Videos zum Thema anschauen [13, 14].

Der vorliegende Beitrag geht vor diesem Hintergrund erstmals systematisch der Frage nach, wie deutschsprachige YouTube‑, Instagram- und TikTok-Videos samt zugehörigen Video-Kommentaren das Thema Menstruation aufgreifen. Ergänzend zu diesem Fokus auf soziale Medien diskutiert der Beitrag am Ende auch, welche Bedeutung Werkzeuge der künstlichen Intelligenz (KI) für die aktuelle und zukünftig Menstruationsaufklärung haben.

### Forschungsstand

Unter *Menstruationsaufklärung* als Teilgebiet der Sexualaufklärung wird die Vermittlung von Informationen über die Menstruation verstanden. Hier kann es sich um formelle Bildungsprozesse durch ausgebildete Fachkräfte oder um das Weitergeben von Erfahrungswissen durch Laien handeln. Die Forschung speziell zu *medialer Menstruationsaufklärung* befasst sich damit, welche Botschaften über die Menstruation auf verschiedenen Medienkanälen verbreitet werden und wie sie beim Publikum ankommen. Die bisherige Forschung zur Menstruationsaufklärung im Internet konzentriert sich dabei teilweise auf das Web, vor allem aber auf soziale Medien, wobei zu beachten ist, dass beide Medienformen sich nicht selten aufeinander beziehen: Webseiten verweisen auf zugehörige Social-Media-Kanäle und in sozialen Medien wird auf Webseiten Bezug genommen.

Die Mehrzahl der Jugendlichen in Deutschland gibt an, Sexualaufklärung neben Elternhaus und Schule vor allem aus dem *Internet* zu beziehen [14]. Wer nach „Menstruation“ oder „Monatsblutung“ googelt, stößt unter den ersten Treffern meist auf Webseiten von Gesundheitsportalen, Krankenkassen, Beratungsstellen, Herstellern von Menstruationsprodukten, Drogerien, Beiträge in Online-Foren und in der Wikipedia [1, 13]. Auch Aufklärungsseiten vom Bundesinstitut für Öffentliche Gesundheit (BIÖG)^2^ werden gefunden sowie Webseiten^3^ und Broschüren zum Download von pro familia, die etwa die Menstruation in leichter Sprache erklären unter dem prägnanten Titel „Blut ist gut!“^4^.

Während eine aktuelle Analyse zu deutschsprachigen *Web-Materialien* zur Menstruationsaufklärung fehlt, zeigt eine Auswertung von 31 englischsprachigen Web-Materialien neben hilfreichen Informationen auch problematische Aspekte: So monieren die Forschenden, dass die Materialien die Menstruation nicht holistisch genug behandeln, sie unzureichend normalisieren und nicht alle verfügbaren Menstruationsprodukte einbeziehen, sondern zu einseitig auf Tampons und Binden fokussieren [15].

Studien zur Repräsentation der Menstruation in *sozialen Medien* konzentrieren sich meist auf Stichproben englischsprachiger Inhalte von einzelnen Plattformen. Identifiziert werden konnten 20 begutachtete Inhaltsanalysen, die Menstruationsdarstellungen und zugehörige Kommentare qualitativ, quantitativ oder computational ausgewertet haben von führenden sozialen Medien wie (alphabetisch):

- Facebook [16],
- Instagram [17–19],
- Reddit [20–22],
- TikTok [23–27],
- Twitter/X [28–31] und
- YouTube [27, 32–35].

Ein Teil der Studien ist dabei eher politisch ausgerichtet und untersucht, inwiefern soziale Medien genutzt werden, um das negative Menstruationsstigma zu überwinden und Menstruationsrechte einzufordern (z. B. [29]). Speziell zum medialen Aktivismus für Menstruationsrechte existiert auch eine erste Buchpublikation [36]. Ein anderer Teil der Studien konzentriert sich auf gesundheitsbezogene Themen im engeren Sinne, etwa auf die Diagnose und Behandlung menstrueller Beschwerden und Störungen (z. B. [34]) sowie zyklusabhängiger Erkrankungen (z. B. Endometriose [37]). In ihrer Bewertung der Menstruationsdarstellungen gehen die Studien auseinander: Viele Inhaltsanalysen betonen den positiven Unterstützungseffekt, der durch einen offenen Austausch über Menstruation in sozialen Medien entsteht (z. B. [16]) und der auch durch Interviewstudien bestätigt wird [38]; andere monieren fortbestehende Negativdarstellungen des Themas (z. B. [19]) sowie die Verbreitung gesundheitsbezogener Fehlinformationen (z. B. [33]).

### Forschungsziel

Vor dem Hintergrund des bisherigen Forschungsstandes und auf Basis eines biopsychosozialen Verständnisses von Menstruationsgesundheit [1] untersucht die vorliegende Studie erstmals systematisch, wie Menstruation in deutschsprachigen Social-Media-Videos auf YouTube, Instagram und TikTok dargestellt wird und wie das Publikum darauf reagiert. Dementsprechend sind folgende Forschungsfragen (F1 bis F4) zu beantworten:

#### F1.

Wer bietet auf YouTube, Instagram und TikTok deutschsprachige Informationsvideos über die Menstruation an?

#### F2.

Welche Inhalte haben diese Informationsvideos?

#### F3.

Welche Qualität haben diese Informationsvideos?

#### F4.

Welche Publikumsreaktionen zeigen sich bei diesen Informationsvideos (Anzahl der Views, Likes und Kommentare sowie Inhalte der Kommentare)?

Das Video-Format und die Plattformen YouTube, Instagram und TikTok wurden wegen ihrer Popularität ausgewählt.

## Methoden

### Untersuchungsdesign

Die Studie wurde im Jahr 2024 als quantitative Querschnittstudie mittels manueller Inhalts- und Qualitätsanalyse durchgeführt [39]. Die Studie ist präregistriert und folgt dem Open-Science-Ansatz, das heißt, die Präregistrierung, die Codebücher, Datensätze, Auswertungsskripte sowie 3 zusätzliche Ergebnistabellen Z1–Z3 sind öffentlich zugänglich (https://osf.io/hyv5g/). Das Untersuchungsmaterial selbst besteht aus öffentlich zugänglichen Social-Media-Videos und zugehörigen öffentlichen Kommentaren, die nach aktuellem Verständnis der Online-Forschungsethik für wissenschaftliche Untersuchungen frei zur Verfügung stehen [40].^5^ Stichprobenbildung, Instrument sowie Datenerhebung und Datenanalyse werden im Folgenden erläutert.

### Stichprobenbildung

Die Studie basiert auf einer Stichprobe von *N* = 500 Videos zur Menstruation, die auf YouTube, Instagram und TikTok veröffentlicht wurden, sowie *N* = 6314 zugehörigen Publikumskommentaren. Die Videos wurden über die Suche nach „Menstruation“ und „Periode“ identifiziert. Es wurden die Top-Videos aus den jeweiligen Plattform-Rankings ausgewählt. Eingeschlossen wurden nur deutschsprachige Videos, die die Menstruation als zentrales Thema sachlich behandeln. Zu jedem Video wurden die 20 meistgelikten Top-Level-Publikumskommentare mit inhaltlichem Bezug zur Menstruation erhoben. Ausgeschlossen wurden Kommentare ohne Menstruationsbezug (z. B. Grüße, Witze, Werbung). Da nicht zu allen Videos 20 themenbezogene Kommentare existierten (sondern im Median 14), ergab sich ein Sample, das kleiner ist als das theoretische Maximalsample von 10.000 Kommentaren. Die Zusammensetzung der Stichproben ist Tab. 1 zu entnehmen. Die Entscheidung, jeweils Top-Videos und Top-Kommentare auszuwählen, basiert auf der Überlegung, dass diese Beiträge und Kommentare die höchste Wahrscheinlichkeit haben, vom Publikum wahrgenommen zu werden.

**Tab. 1:** Zusammensetzung der Stichprobe von Top-Videos zur Menstruation auf YouTube, Instagram und TikTok und zugehörigen themenbezogenen Kommentaren

	YouTube	Instagram	TikTok
**Anzahl der Top-Videos (*N*** **=** **500)**	**150**	**150**	**200**^**a**^
*Suchbegriff(e)*	Menstruation; Periode	Menstruation; Periode	Menstruation; Periode
*Auswahl der Videos*	„Inkognito“ Auswahl nach Reihenfolge der von YouTube präsentierten deutschsprachigen Suchergebnisse nach YouTubes Relevanz ohne Veränderung der Filtereinstellungen	Ein neuer Forschungsaccount wurde erstellt. Auswahl nach Reihenfolge der von Instagram präsentierten deutschsprachigen Suchergebnisse unter der „Für-dich“-Kategorie via Android-Emulator „BlueStacks“	Ein neuer Forschungsaccount wurde erstellt. Auswahl nach Reihenfolge der von TikTok präsentierten deutschsprachigen Suchergebnisse ohne Veränderung der Filtereinstellungen
*Video-Länge in Minuten*			
(Min) 00:03	00:17	00:03	00:05
(Max) 29:57	29:57	12:40	03:52
(Mittel) 02:51	07:40	00:56	00:41
(Median) 00:52	05:38	00:36	00:32
**Anzahl der Top-Kommentare (*N*** **=** **6314)**	**1683**	**1619**	**3012**
*Auswahl der Kommentare*	Top 20 meistgelikte Kommentare mit Menstruationsbezug pro ausgewähltem Video	Top 20 meistgelikte Kommentare mit Menstruationsbezug pro ausgewähltem Video	Top 20 meistgelikte Kommentare mit Menstruationsbezug pro ausgewähltem Video
*Kommentar-Länge in Anzahl der Wörter*			
(Min) 1	1	1	1
(Max) 1192	1192	313	99
(Mittel) 23	40	28	11
(Median) 14	25	19	9

^a^Größeres Teilsample für TikTok-Videos wegen ihrer Kürze

### Instrument

Zur Beantwortung der 4 Forschungsfragen wurde ein Codebuch für die Videos und eines für die Kommentare entwickelt, teils induktiv anhand des Materials, teils deduktiv anhand der Fachliteratur. Zudem wurde bei der Entwicklung und Validierung des Codebuchs auf die Expertise einer gynäkologischen Fachärztin (Autorin 3) zurückgegriffen. Das Video-Codebuch gliedert sich in folgende 5 Teile:

1. *Formale Variablen*: Sie erfassen allgemeine Merkmale der untersuchten Videos (z. B. Titel des Videos, Veröffentlichungsdatum, Videolänge).
2. *Typ des Video-Anbieters*: Zur Beantwortung von F1 wurden verschiedene Anbietertypen differenziert. Besonders wichtig war hier gemäß der Fachliteratur zur Online-Gesundheitskommunikation (z. B. [41]), ob Menstruationsvideos von Medienprofis, Gesundheitsprofis oder Gesundheitslaien stammen.
3. *Video-Inhalte*: Zur Beantwortung von F2 wurde erfasst, welche Aspekte der Menstruation im Video vorkommen [1], nämlich (1) Menstruation und Zyklus (z. B. physiologische Prozesse), (2) Menstruationserleben (z. B. Schmerzerleben), (3) Menstruationsmanagement (z. B. Nutzung von Menstruationsprodukten), (4) medizinische Menstruationsversorgung (z. B. Behandlung menstrueller Störungen), (5) Menstruation im Kontext (z. B. Menstruation beim Sport) sowie (6) gesellschaftlicher Umgang (z. B. Menstruationsaktivismus). Zudem wurde erfasst, welche Art von Wissen präsentiert wird (Fakten- und/oder Erfahrungswissen) und ob die Darstellung der Menstruation eine wertende Tendenz hat (negativ, positiv, ambivalent, neutral; [41]).
4. *Inhaltsqualität* der Videos: Zur Beantwortung von F3 wurden die Videos mit dem mDISCERN-Index beurteilt [42], dem meistgenutzten Instrument zur Messung der Qualität von Social-Media-Gesundheitsinformationen. Der Index beinhaltet 5 Qualitätskriterien: 1. die Nennung der Ziele des Beitrags, 2. die Verwendung von zuverlässigen Informationsquellen, 3. die ausgewogene und unvoreingenommene Informationsdarstellung, 4. die Angabe von weiterführenden Informationen und 5. die Nennung von Kontroversen oder Unsicherheiten. Die Beurteilung der Einzelkriterien fließt in einen mDISCERN-Gesamtwert ein mit einem Wertebereich von 0 (schlechteste Qualität) bis 5 (beste Qualität).
5. *Quantitative Publikumsreaktionen*: Zur Beantwortung von F4 wurden für jedes Video die Anzahl a) der Views, b) der Likes und c) der Kommentare zum Erhebungsdatum (17.10.2024–06.11.2024) erfasst. Diese Social-Media-Metriken werden unter dem jeweiligen Social-Media-Beitrag angegeben und zeigen, wie stark das Publikum mit dem Beitrag interagiert.

Das Codebuch für die Video-Kommentare gliedert sich in 2 Blöcke:

1. *Formale Variablen:* Sie erfassen, auf welches Video sich der Kommentar bezieht, und dokumentieren den Wortlaut des Kommentars.
2. *Qualitative Publikumsreaktionen: *Themenbezogene Kommentare wurden nach Art des Kommentars codiert (z. B. Rückfrage, Teilen eigener Erfahrungen; [41]).

Die Reliabilität der Kategorien wurde anhand von 50 aus dem Sample zufällig ausgewählten Videos (Video-Codebuch) bzw. 300 zufällig ausgewählten Kommentaren (Kommentar-Codebuch) von 2 geschulten unabhängigen Codierenden erfasst. Berechnet wurde der im Feld der Medieninhaltsforschung etablierte Reliabilitätskoeffizient Gwets AC1, der mit Mittelwerten von 0,91 für Videos und 0,87 für Kommentare auf gute Messgenauigkeit hinweist ([43]; Zusatztabelle Z1 mit allen Reliabilitätskoeffizienten ist https://osf.io/hyv5g/ zu entnehmen).

### Datenerhebung und Datenanalyse

Die Datenerhebung erfolgte im Rahmen manueller Codierung durch 2 geschulte Codierende. Grundlage der Codierung waren dabei die beiden oben dargestellten reliabilitätsgeprüften Codebücher. Die Datenanalyse erfolgte deskriptiv- und inferenzstatistisch (Häufigkeitsanalysen, Chi-Quadrat-Tests bzw. Fishers exakte Tests und Varianzanalysen) unter Nutzung der Software R (R-Pakete DescTools, expss, stats, irrCAC).

## Ergebnisse

### Anbietertypen von Menstruationsvideos in sozialen Medien (F1)

Auf sozialen Medien als Mitmach-Plattformen äußern sich in erster Linie Gesundheitslaien zur Menstruation; dieser Anbietertyp dominiert vor allem unter den Top-Videos auf TikTok (57 %; Tab. 2). Ein Beispiel ist das 57-sekündige TikTok-Video „Habt ihr vor eurer Periode PMS?“ der Influencerin Leonie Schley („JustLeo“), in dem sie Körperfunktionen und Organe als sprechende Figuren auftreten lässt.^6^ Auf YouTube sind dagegen Medienprofis als Anbieter der Top-Menstruationsvideos (31 %) signifikant stärker vertreten als auf den anderen beiden Plattformen. Ein Beispiel ist das 25-minütige YouTube-Video „Kein Geld für Tampons: Warum die Periode in Armut zu einem Problem wird“ des Reportage-Formats Y‑Kollektiv (Radio Bremen): Die Reporterin begleitet Betroffene, unter anderem eine junge Frau in Deutschland, die im Wohnwagen lebt und für Binden betteln muss, sowie Schülerinnen in Kenia, die ihre Periode mangels Hygieneprodukten zu Hause aussitzen.^7^ Weiterhin sind Gesundheitsprofis aus Gynäkologie oder Sexualpädagogik (17 %) sowie Unternehmen wie Hersteller von Tampons oder Zyklus-Apps (15 %) mit eigenen Menstruationsvideos auf allen 3 Plattformen nennenswert sichtbar.

**Tab. 2:** Anbietertypen der Top-Videos zur Menstruation auf YouTube, Instagram und TikTok (Anteile in Prozent der Videos im Sample)

Anbietertyp^a^	Gesamt *N* = 500	YouTube *n* = 150	Instagram *n* = 150	TikTok *n* = 200	*p*
Gesundheitslaie	42,4	28,0	38,0	56,5	< 0,001
Medienprofi	21,4	31,3	24,7	11,5	< 0,001
Gesundheitsprofi	17,0	20,0	18,0	14,0	0,311
Unternehmen	15,2	12,7	16,7	16,0	0,578
Religionsvertreter ^b^	1,8	2,7	1,3	1,5	0,698
Sonstiges/Unklar	1,2	4,0	0,0	0,0	/
Politikakteur ^b^	1,0	1,3	1,3	0,5	0,623

Prozentwerte basieren auf den Top-Videos zur Menstruation und sind nach den Werten in der Spalte Gesamt absteigend sortiert. Zeilenweise Auswertung mit 2‑dimensionalen Chi-Quadrat-Tests, *df* = 2

^a^Gesamttest: 2‑dimensionaler Chi-Quadrat-Test (Anbieter-Typ × Plattform), *χ*^*2*^(12) = 52,83, *p* < 0,001, Cramérs *V* = 0,23

^b^Aufgrund niedriger Zellenbesetzungen wurde Fishers exakter Test gerechnet

### Inhalte von Menstruationsvideos in sozialen Medien (F2)

Inhaltlich beschäftigen sich die meisten Menstruationsvideos mit dem Management der Blutung etwa durch verschiedene Menstruationsprodukte (60 %) sowie mit dem Erleben von Schmerzen und anderen Beschwerden (58 %, F2). Knapp jedes vierte Top-Menstruationsvideo (23 %) geht auch auf gesellschaftliche Aspekte wie Menstruationsaktivismus ein; die längeren YouTube-Videos sprechen dabei häufiger 3 oder mehr verschiedene Themen an im Gegensatz zu den kürzeren Instagram- oder TikTok-Videos (Tab. 3). Dabei kann auch ein kurzer TikTok-Clip^8^ von nur 55 s wichtige Informationen liefern, indem er normalisierend darstellt, dass man mit Tampon, Menstruationstasse oder Menstruationsbadekleidung (Thema: Menstruationsmanagement) problemlos schwimmen gehen kann (Thema: Menstruation im Kontext, hier: Sport). Die Häufigkeitsverteilung von Unterthemen ist Zusatztabelle Z2 unter https://osf.io/hyv5g/ zu entnehmen. Hinsichtlich der Art der präsentierten Informationen zeigt sich grob eine Dreiteilung zwischen reinem Erfahrungswissen (37 %), reinem Faktenwissen (27 %) und einer Kombination aus beidem (36 %).

**Tab. 3:** Themen der Top-Videos zur Menstruation auf YouTube, Instagram und TikTok (Anteile in Prozent der Videos im Sample)

Thematische Aspekte der Menstruation	Gesamt	YouTube	Instagram	TikTok
	%	%	%	%
Menstruationsmanagement (z. B. Menstruationsprodukte)	60,2	74,0	54,7	54,0
Menstruationserleben (z. B. Beschwerden wie Schmerzen)	58,2	73,3	58,7	46,5
Menstruation im Kontext (z. B. Menstruation beim Sport)	39,4	63,3	31,3	27,5
Menstruation und Zyklus (z. B. physiologische Prozesse)	30,8	54,0	21,3	20,5
Medizinische Menstruationsversorgung (z. B. Diagnose und Behandlung von menstruellen Störungen)	27,8	48,0	24,7	15,0
Gesellschaftlicher Umgang (z. B. Menstruationsaktivismus)	23,0	39,3	20,7	12,5

*N* = 500 Videos (YouTube: 150; Instagram: 150; TikTok: 200). Die Prozentwerte sind nach den Werten in der Gesamtspalte absteigend sortiert. Da ein Video mehrere Themen behandeln kann, überschreiten die Summen 100 %. 2‑dimensionaler Chi-Quadrat-Test zwischen Plattform und Anzahl der behandelten Themen (1, 2 oder 3+ Themen), *χ*^*2*^(4) = 128,56, *p* < 0,001, Cramérs *V* = 0,36

**Abb. 1:**
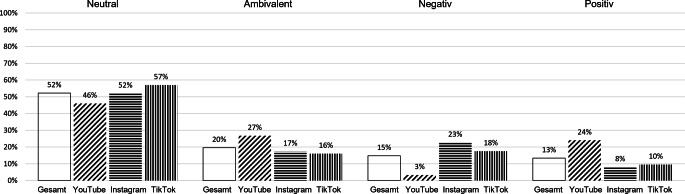
Wertende Darstellung in den Top-Videos zur Menstruation auf YouTube, Instagram und TikTok (Anteile in Prozent der Videos im Sample). *Anmerkung*: Prozentwerte basieren auf *N* = 500 Videos (YouTube: 150; Instagram: 150; TikTok: 200) in absteigender Reihenfolge der Häufigkeiten der Wertungen. 2‑dimensionaler Chi-Quadrat-Test (Bewertung x Plattform), *χ*^*2*^(6) = 46,22, *p* < 0,001, *V* = 0,21

Es existieren unter den Top-Videos solche, deren Inhalte insgesamt eine negative Wertung (15 %) vermitteln, die beispielsweise nur auf Beschwerden und Leiden eingehen, ebenso wie solche mit positiver Wertung (13 %), die beispielsweise die Menstruation als Ausdruck von Weiblichkeit feiern (Abb. 1). Weiterhin gibt es ambivalente Videos, die positive und negative Bewertungen enthalten (20 %). Dominierend sind jedoch neutrale Videos (52 %), die eine ausgewogene und sachliche Informationsvermittlung betreiben. Ein Beispiel dafür ist das YouTube-Video „Braun, rot, rosa? Was ist normal bei Periodenblut | Mythen und Fakten rund um die Periode | Blutung“ des Kanals „Gynäko.Logisch“ (8:15 min). In diesem Video informiert der Gynäkologe Dr. med. Konstantin Wagner sachlich in Form von Faktenwissen über verschiedene Aspekte der Monatsblutung und erklärt unter anderem, warum das Blut manchmal heller und manchmal dunkler aussieht oder sich auch Klümpchen bilden können.^9^

### Qualität von Menstruationsvideos in sozialen Medien (F3)

**Abb. 2:**
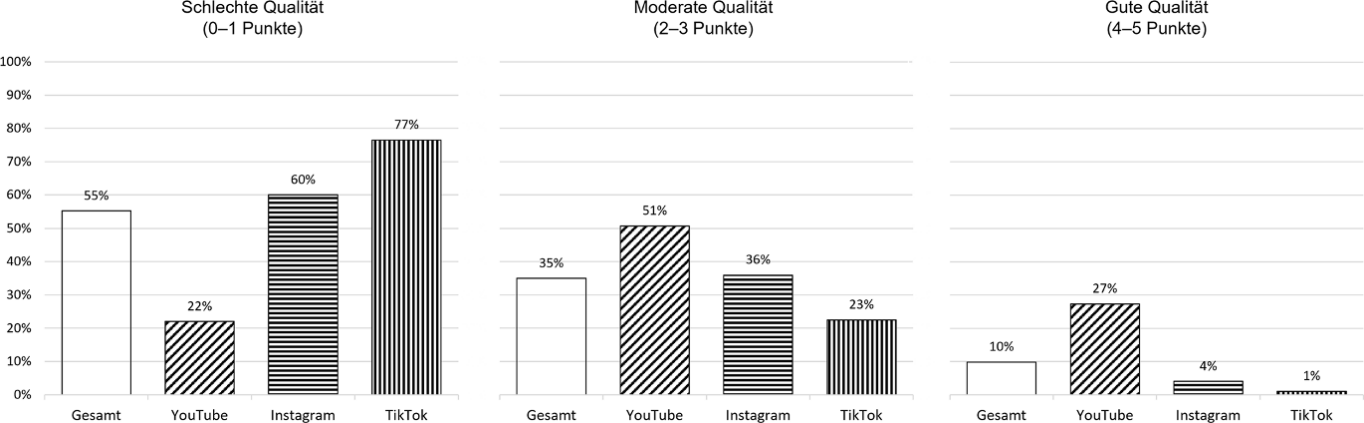
Qualität der Top-Videos zur Menstruation auf YouTube, Instagram und TikTok (gemessen mit dem mDISCERN-Index). *Anmerkung*: Prozentwerte zur Informationsqualität basieren auf *N* = 500 Videos (YouTube: 150; Instagram: 150; TikTok: 200), gemessen mit dem mDISCERN-Index. Der mDISCERN-Gesamtwert ergibt sich aus der Summenpunktzahl der 5 Bewertungsfragen. 2‑dimensionaler Chi-Quadrat-Test (Informationsqualität × Plattform), *χ*^*2*^(4) = 134,51, *p* < 0,001, Cramérs *V* = 0,37

Gemessen mit dem mDISCERN-Index haben die deutschsprachigen Top-Videos zur Menstruation mehrheitlich (55 %) eine schlechte Informationsqualität, 35 % erreichen eine moderate und nur 10 % eine gute Qualität (Abb. 2). Dabei zeigen sich deutliche Differenzen zwischen den Plattformen (2-dimensionaler Chi-Quadrat-Test: *p* < 0,001): YouTube übertrifft in der Inhaltsqualität Instagram und TikTok, da YouTube-Videos gemäß den Kriterien des mDISCERN-Index häufiger auf zuverlässige Quellen verweisen, weiterführende Informationen angeben und eine ausgewogene Darstellung der Menstruation anbieten. So erzielte denn auch das im vorigen Abschnitt beschriebene YouTube-Video vom Kanal „Gynäko.Logisch“ den vollen Punktwert von 5 auf der mDISCERN-Skala.

### Publikumsreaktionen auf Menstruationsvideos in sozialen Medien (F4)

**Abb. 3:**
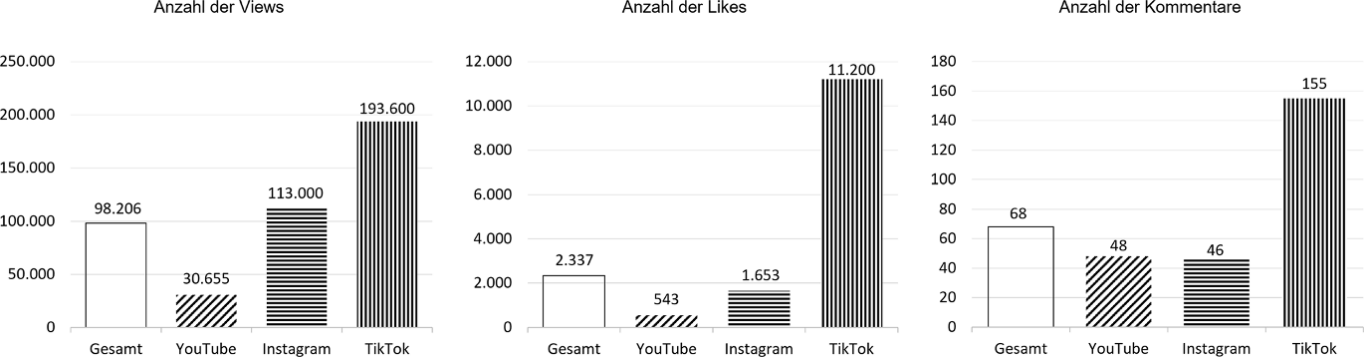
Publikationsreaktionen auf die Top-Videos zur Menstruation auf YouTube, Instagram und TikTok (Medianwerte). *Anmerkung*: Dargestellt werden die Medianwerte der Social-Media-Metriken von insgesamt *N* = 500 Videos zur Menstruation (YouTube: 150; Instagram: 150; TikTok: 200). Median der *Views* pro Video; Median der *Likes* pro Video, Median der *Kommentare* pro Video

Die untersuchten deutschsprachigen Top-Videos zur Menstruation zeigen bedeutsame Publikumsreichweiten: Der Median liegt bei rund 98.000 Aufrufen pro Video. Die oben genannte gesellschaftskritische Reportage „Kein Geld für Tampons: Warum die Periode in Armut zu einem Problem wird“ aus dem Jahr 2019 hat bislang über 1,4 Mio. Views gesammelt (Stand: Oktober 2025) – dabei ist sie als Top-Video mehrere Jahre nach Veröffentlichung immer noch relevant, das heißt, sie wird auch heute weiterhin geschaut und kommentiert. Noch größere Interaktionsraten als YouTube und Instagram erreicht jedoch TikTok: Hier erlangen die Top-Menstruationsvideos mit Abstand die meisten Views, Likes und Kommentare (Abb. 3).

Betrachtet man die Inhalte der meistgelikten themenbezogenen Kommentare, so zeigt sich, dass diese ganz überwiegend dem Teilen eigener Erfahrungen des Publikums gewidmet sind (63 %), zuweilen auch Nachfragen (14 %) enthalten (für Details zu den Inhalten der Kommentare siehe Zusatztabelle Z3 unter https://osf.io/hyv5g/). Zu dem eingangs erwähnten TikTok-Video zum prämenstruellen Syndrom (PMS), in dem die Influencerin „JustLeo“ verschiedene Beschwerden (Heißhunger, Gereiztheit, Müdigkeit, Verdauungsprobleme usw.) im Rollenspiel humorvoll inszeniert, sind seit Veröffentlichung im Jahr 2023 über 1600 Kommentare eingegangen. Manche fragen nach, was „PMS“ bedeutet, worauf die Videomacherin kommentiert: „Als prämenstruelles Syndrom bezeichnet man ein Bündel aus körperlichen und psychischen Beschwerden, die einige Tage vor der Periode auftreten können.“ Viele teilen eigene PMS-Erfahrungen („bei mir kommt noch Übelkeit dazu“) und freuen sich, dass das Thema so offen und gleichzeitig witzig angesprochen wird („Liebe deine Videos 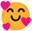

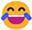
 triffst es immer auf den Punkt“).

## Diskussion

### Interpretation der Befunde

Die vorliegende Studie zeigt, dass die reichweitenstärksten deutschsprachigen Menstruationsvideos auf YouTube, Instagram und TikTok überwiegend von Gesundheitslaien stammen, gefolgt von Medienprofis (F1). Gesundheitsprofis stehen aber immerhin an dritter Stelle und sind auf allen 3 untersuchten Plattformen mit Top-Videos sichtbar, ähnlich wie das auch bei Verhütungsthemen in sozialen Medien der Fall ist [41]. Hier zeigt sich, dass es durchaus Erfolgsmodelle gibt, wie Gesundheitsprofis ihre Expertise plattformgerecht erfolgreich auf Social Media platzieren und damit einer oft formulierten Forderung an moderne Gesundheitsaufklärung gerecht werden, nämlich dahin zu gehen, wo die Jugendlichen sind [23, 44].

Die Inhalte der Menstruationsvideos decken unterschiedliche Facetten der Menstruationsgesundheit [44] ab (F2). Im Vordergrund stehen dabei aber nicht politische und aktivistische Aspekte, auf die sich einige internationale Vorgängerstudien beziehen [29] und die auch im vorliegenden Datensatz auftauchen (z. B. Menstruationsarmut), sondern praktische Fragen (z. B. Nutzung von Menstruationsprodukten) sowie das Erleben von Schmerzen und anderen Beschwerden, die anhand von Erfahrungs- und Faktenwissen behandelt werden. Angesichts tradierter Stigmatisierung der Monatsblutung ist es als hilfreich einzuschätzen, dass das Thema auf den 3 führenden Social-Media-Plattformen so sichtbar und damit öffentlich besprechbar geworden ist.

Die Top-Menstruationsvideos schneiden indessen nicht gut ab, wenn sie mit dem mDISCERN-Index bewertet werden, dem etabliertesten Maß für qualitätvolle Online-Gesundheitsinformationen (F3). Doch dieser Negativbefund, der sich mit früheren Studien deckt [33, 41], muss eingeordnet werden: Die geringen Qualitätswerte sind nicht gleichbedeutend mit Falschinformationen, sondern entstehen vor allem dadurch, dass Quellenangaben und Verweise auf Hintergrundinformationen oft fehlen. Diese sind jedoch bei Instagram und TikTok plattformtechnisch kaum platzierbar, während YouTube-Videos mit einer ausführlichen textlichen Videobeschreibung versehen werden können. Darüber hinaus berücksichtigt der mDISCERN-Index nur Faktenwissen und ignoriert damit Funktionen und Nutzen von subjektivem Erfahrungswissen (z. B. sich im eigenen Erleben verstanden fühlen).

Die große Zahl der Views, Likes und Kommentare der Top-Menstruationsvideos belegt im Einklang mit früheren Inhaltsanalysen [41] sowie mit Befragungsstudien [38, 45] die große Relevanz sozialer Medien für die Sexualaufklärung (F4). Zu beachten ist auch, dass die Kommentarspalten zu einem lebendigen Ort des offenen Erfahrungsaustauschs geworden sind. Auch und gerade subjektive Videos, die Erfahrungswissen spiegeln und emotional gefärbt sind, mögen das Publikum zum Mitdiskutieren einladen und somit die Sprechfähigkeit zu tabuisierten Themen fördern sowie ein Gefühl von Gemeinschaft und wechselseitiger Unterstützung vermitteln [34].

### Limitationen

Soziale Medien sind dynamisch, die Studie bietet daher nur eine Momentaufnahme. Künftige Forschung sollte den Diskurs im Zeitverlauf beobachten und weitere Social-Media-Plattformen (z. B. Facebook, X/Twitter, Reddit, Twitch) in die Vergleiche einbeziehen. Qualitätsindikatoren gilt es weiterzuentwickeln, da der mDISCERN-Index einseitig auf Faktenwissen ausgerichtet ist und die Qualität von geteiltem subjektiven Erfahrungswissen ignoriert. Ebenso berücksichtigt der Index nicht Faktoren wie Unterhaltungswert der Videos oder Besonderheiten einzelner Social-Media-Plattformen (z. B. textliche Video-Beschreibungen). Ergänzend zum inhaltsanalytischen Zugang können Befragungsstudien mit verschiedenen Anbietertypen sowie mit dem Publikum von Menstruationsvideos zusätzliche Einblicke in menstruationsbezogene Kommunikationsziele und subjektive Wahrnehmungen und Wirkungen der Videos liefern.

## Fazit und Ausblick

Die vorliegende Inhalts- und Qualitätsanalyse beschreibt die Menstruationskommunikation auf YouTube, Instagram und TikTok. Dabei bietet YouTube im Plattformenvergleich die gehaltvollsten Videos, meist Presse-Reportagen, und auch die längsten Kommentare. Hier werden am ehesten verschiedene Aspekte des Themas im Zusammenhang angesprochen. Auf Instagram dominiert die Darstellung des subjektiven Menstruationserlebens, teils negativ als persönliches Leiden, teils positiv als Bestätigung von weiblicher Identität oder als Thema des feministischen Aktivismus [1]. Auf TikTok bekommt ein sehr junges Publikum knappe Clips vom Algorithmus zugespielt, was oftmals zum Teilen eigener Erfahrungen in den Kommentarspalten führt. Für die professionelle Sexualaufklärung ergeben sich die beiden Anforderungen, a) mit hochwertigem eigenen Content präsent zu sein sowie b) durch zeitgemäße sexualbezogene Medienbildung die Social-Media-Nutzenden zu befähigen, mit Videos und Video-Kommentaren zur Menstruation zielgerichtet und kritisch umzugehen.

Das Social-Media-Zeitalter transformiert sich aktuell zum Zeitalter der künstlichen Intelligenz (KI). Jugendliche suchen Sexual- und Menstruationsaufklärung zunehmend bei KI-Chatbots wie ChatGPT, Gemini oder Claude [46, 47]. Inhaltsanalysen von KI-Output zeigen bislang eine recht hohe Informationsqualität, sowohl was sachliche Korrektheit als auch Orientierung an Menschenrechten betrifft [48, 49]. Weitgehend unklar ist bislang jedoch, wie eine KI-gestützte Sexualaufklärung in Zukunft aussehen wird: Wie werden Fachkräfte KI-Tools nutzen, um neue textliche und bildliche Inhalte (beispielsweise zur Menstruation) für ihre Flyer, Webseiten und Social-Media-Auftritte zu generieren? Und wie werden sich Informations- und Ratsuchende an die KI wenden, ihre Anfragen formulieren und die erhaltenen KI-Antworten interpretieren und einordnen und diese Erfahrungen wiederum auf sozialen Medien besprechen (siehe z. B. Hashtag #ChatGPT auf TikTok)? Ähnlich wie bei sozialen Medien gehen auch bei KI-Werkzeugen die öffentlichen und fachlichen Einschätzungen auseinander: Während die einen vor Fehlinformationen durch KI warnen, sehen andere KI-Tools als sinnvolle Unterstützung für Sexualaufklärung und Selbstbestimmung [50]. Voraussetzung ist hier neben der verantwortungsvollen Gestaltung der KI-Modelle vor allem auch die Stärkung der KI-Kompetenz der Nutzenden.

## Data Availability

Die Studie ist präregistriert und folgt dem Open-Science-Ansatz, das heißt, alle Codebücher, Datensätze, Auswertungsskripte sowie zusätzliche Ergebnistabellen Z1–Z3 sind auf dem Server des Open Science Framework (OSF) öffentlich zugänglich hinterlegt: https://osf.io/hyv5g/.

## Footnotes

1 Im vorliegenden Beitrag benutzen wir im Einklang mit der Fachliteratur sowohl geschlechtsspezifische Bezeichnungen (Mädchen, Frauen), um die Relevanz der Menstruation für Frauengesundheit und Frauenrechte zu betonen, als auch geschlechterinklusive Bezeichnungen (z. B. Menschen mit Gebärmutter; Menstruierende; engl. „menstruators“), um menstruierende nichtbinäre Personen oder Transmänner einzuschließen.

2 Menstruationsaufklärung im Web vom BIÖG: https://www.frauengesundheitsportal.de/themen/gutartige-gebaermuttererkrankungen/schmerzhafte-starke-unregelmaessige-regelblutungen/ (letzter Zugriff: 06.10.2025).

3 Menstruationsaufklärung im Web von pro familia: https://www.profamilia.de/fuer-jugendliche/pubertaet/regelblutung (letzter Zugriff: 06.10.2025).

4 Informationsbroschüre zur Menstruation in leichter Sprache vom pro familia Landesverband Sachsen im Web-Download: https://www.profamilia.de/fileadmin/landesverband/lv_sachsen/Melisse/2025_pro-familia-Blut_ist_gut.pdf (letzter Zugriff: 06.10.2025).

5 Das gilt für unsere Studie insbesondere deswegen, weil die Videos von reichweitenstarken Accounts stammen, die sich ihrer Öffentlichkeitswirkung bewusst sind, und da wir Video-Kommentare nur anonym zitieren.

6 https://www.tiktok.com/@justleo/video/7201573297681763590 (letzter Zugriff: 06.10.2025).

7 https://www.youtube.com/watch?v=wZgSymcGS3Q (letzter Zugriff: 06.10.2025).

8 https://www.tiktok.com/@wahrscheinlichpeinlich/video/7068545761599933701 (letzter Zugriff: 06.10.2025).

9 https://www.youtube.com/watch?v=QICaD1zOEzQ (letzter Zugriff: 06.10.2025).
